# Construction of an improved *Aspergillus niger* platform for enhanced glucoamylase secretion

**DOI:** 10.1186/s12934-018-0941-8

**Published:** 2018-06-16

**Authors:** Markus R. M. Fiedler, Lars Barthel, Christin Kubisch, Corrado Nai, Vera Meyer

**Affiliations:** Department Applied and Molecular Microbiology, Institute of Biotechnology, Technische Universität Berlin, Gustav-Meyer-Allee 25, 13355 Berlin, Germany

**Keywords:** *Aspergillus niger*, Tet-on, Protein secretion, GTPase RacA, v-SNARE, Hyperbranching, Post-Golgi carrier, Glucoamylase

## Abstract

**Background:**

The lifestyle of filamentous fungi depends on the secretion of hydrolytic enzymes into the surrounding medium, which degrade polymeric substances into monomers that are then taken up to sustain metabolism. This feature has been exploited in biotechnology to establish platform strains with high secretory capacity including *Aspergillus niger*. The accepted paradigm is that proteins become mainly secreted at the tips of fungal hyphae. However, it is still a matter of debate if the amount of growing hyphal tips in filamentous fungi correlates with an increase in secretion, with previous studies showing either a positive or no correlation.

**Results:**

Here, we followed a systematic approach to study protein secretion in *A. niger*. First, we put the *glaA* gene encoding for glucoamylase (GlaA), the most abundant secreted protein of *A. niger*, under control of the tunable Tet-on system. Regulation of *glaA* gene expression by omitting or adding the inducer doxycycline to cultivation media allowed us to study the effect of *glaA* under- or overexpression in the same isolate. By inducing *glaA* expression in a fluorescently tagged v-SNARE reporter strain expressing GFP-SncA, we could demonstrate that the amount of post-Golgi carriers indeed depends on and correlates with *glaA* gene expression. By deleting the *racA* gene, encoding the Rho-GTPase RacA in this isolate, we generated a strain which is identical to the parental strain with respect to biomass formation but produces about 20% more hyphal tips. This hyperbranching phenotype caused a more compact macromorphology in shake flask cultivations. When ensuring continuous high-level expression of *glaA* by repeated addition of doxycycline, this hyperbranching strain secreted up to four times more GlaA into the culture medium compared to its parental strain.

**Conclusion:**

The data obtained in this study strongly indicate that *A. niger* responds to forced transcription of secretory enzymes with increased formation of post-Golgi carriers to efficiently accommodate the incoming cargo load. This physiological adaptation can be rationally exploited to generate hypersecretion platforms based on a hyperbranching phenotype. We propose that a *racA* deletion background serves as an excellent chassis for such hypersecretion strains.

**Electronic supplementary material:**

The online version of this article (10.1186/s12934-018-0941-8) contains supplementary material, which is available to authorized users.

## Background

Filamentous fungi follow a foraging feeding behaviour. During growth, they actively search for food through their intricate hyphal network and nutrients are acquired by extracellular digestion of complex polymers such as plant polysaccharides [1]. The tip of a growing fungal hypha is supposed to be the most active region of protein secretion and a strong correlation between sustained polarised growth and protein secretion at the hyphal tip is generally accepted [2–5]. However, investigations on the fungal cell factories *Aspergillus niger* and *A. oryzae* gave contradictory results while assessing a direct link between amount of active fungal hyphae and secretion, with previous studies showing either a positive correlation [6, 7] or none [7]. Similarly, despite four decades of studying protein secretion in filamentous fungi, our understanding about protein trafficking and secretion is still limited, thus hampering the rational establishment of hypersecretion platform strains for biotechnological purposes [8, 9]. Given the outstanding secretory capacities of filamentous species such as *A. niger* and their promises as superior platform strains cultivable on renewable lignocellulosic feedstocks, it is of great interest to fully understand and exploit the link between polarised growth and secretion.

Hyphal growth is characterised by apical extension. This is ensured by polarised exocytosis of biosynthetic enzymes and their substrates eventually expanding the cell membrane and cell wall [10–13]. A central hub of the conventional secretory pathway is the nonstacked fungal Golgi because it sorts protein cargoes either to the plasma membrane, the endovascular system or the extracellular space (for a comprehensive review, the reader is directed to an excellent recent paper [13]). In brief, coat protein complex II (COPII)-coated vesicles bud off from the endoplasmic reticulum (ER) and coalesce with early Golgi cisternae, which ensure protein glycosylation. Golgi cisternae progressively change their protein and lipid content thus becoming enriched in cargo and eventually maturing to late Golgi cisternae (also called *trans*-Golgi network, TGN). In *A. nidulans*, it was shown that the TGN cisternae finally maturate to post-Golgi carriers by recruiting RabE and engaging motor proteins [14, 15]. The latter assist in movement of post-Golgi secretory vesicles toward the hyphal apex along microtubules (long-distance transport via kinesin-1 or kinesin-3) and actin filaments (myosin-5-mediated final transport to the plasma membrane) [16]. However, before secretory vesicles undergo fusion with the plasma membrane, they accumulate at the hyphal tip and become visible in a structure called Spitzenkörper [17]. Most recently, it was discovered that secretory vesicles accumulate at the Spitzenkörper in a pulsatory way, mediating a stepwise extension of the hyphal tip [16]. This observation is congruent with a pulsatory calcium influx which controls actin polymerization and exocytosis [18]. Hence, secretory vesicles are retained in the Spitzenkörper before being tethered to the plasma membrane. By molecular interactions between SNARE (soluble *N*-ethylmaleimide-sensitive factor attachment protein receptor) proteins, the post-Golgi cargoes become released into the extracellular space or embedded into the growing plasma membrane, thus either secreting enzymes or providing the enzymes required for cell wall expansion [13]. Several SNARE proteins were reported to be involved in fusion steps involving retrograde and anterograde vesicular transport between the ER and the Golgi as well as in fusion steps involving endosomal or vacuolar transport [19, 20]. During vesicle fusion, the α-helix of a monomeric vesicular-SNARE (v-SNARE) in post-Golgi secretory vesicles interacts with three α-helices of an oligomeric target-SNARE (t-SNARE) in the plasma membrane, forming the trans-SNARE complex [21–23]. This triggers fusion of the vesicle with the target membrane, forming the cis-SNARE complex, followed by ATP-dependent SNARE complex dissociation [24]. Calcium functions as a regulator of vesicle fusion; however, not all SNARE-mediated fusion steps in the secretory pathway are calcium-dependent [25]. In *S. cerevisiae*, the v-SNAREs Snc1p and its paralog Snc2p locate to post-Golgi secretory vesicles [21, 26] conferring fusion of the post-Golgi carrier with the plasma membrane via the interaction with the membrane-localized t-SNAREs proteins Sso1p and its paralog Sso2p [27, 28]. Studies that analysed the localization of orthologs of Snc1p in various filamentous fungi including *A. niger* revealed a highly polarised accumulation of AoSnc1 (*A. oryzae*), SncA (*A. niger*), SynA (*A. nidulans*) and SYN-1 (*Neurospora crassa*) at the tip of growing hyphae [2, 29–31]. In all likelihood, they are a component of post-Golgi secretory vesicles in filamentous fungi and become a transient component of the plasma membrane when exocytosis occurs, but are thereafter recycled by the sub-apical endocytic ring. It is thought that this involves post-Golgi sorting endosomes which ensure SynA/Snc1-containing membranes to be transported along microtubules back to the TGN where they eventually fuse with new cargo-loaded post-Golgi carriers [2]. Hence, endocytotic recycling processes are essential for maintaining hyphal polarity in filamentous fungi [13].

In *A. niger*, we could demonstrate that apical dominance in young and mature hyphae of *A. niger* is also mainly controlled by the Rho GTPase RacA, thought to mediate actin polymerization and depolymerisation at the hyphal apex [32]. The *A. niger* Rho GTPases RacA and CftA (Cdc42p) can substitute each other with respect to actin polymerization at the hyphal tip, but actin depolymerisation is secured by RacA and not by CftA. Hence, a Δ*racA* strain is impaired in actin disassembly and in consequence frequently loses apical dominance thus provoking a hyperbranching phenotype [32]. Notably, this hyperbranching phenotype was paralleled by reduced GFP-SncA accumulation at hyphal tips, although physiological profiles gathered from controlled bioreactor cultivations of the Δ*racA* and its wildtype strain uncovered that their growth curves, maximum specific growth rates and specific protein secretion rates were nearly identical. We thus hypothesized that the same amount of secretory vesicles is merely distributed to more tips in the Δ*racA* strain, resulting in less secretory vesicles per individual tip, and that the capacity of the hyphal tip growing apparatus to accommodate vesicles is therefore—at least in the Δ*racA* strain—not fully exploited [35]. To refute or verify this hypothesis, we challenged in the current study the Δ*racA* strain to overexpress one of its homologous and abundantly secreted proteins by putting it under conditional transcriptional control of the Tet-on system [33]. We selected glucoamylase (glucan 1,4-α-glucosidase, GlaA) as model protein as this is the major secreted protein of *A. niger* (up to 30 g/L [34]) with important implications for the food and biofuel industry [8]. By using a GFP-SncA labelled reporter strain as background strain, we show here that (i) more post-Golgi carriers accumulate at hyphal tips in both Δ*racA* and its parental strain upon Tet-on driven overexpression of the *glaA* gene, and that (ii) this specifically leads to an increased glucoamylase secretion in the hyperbranching Δ*racA* strain. Our study thus validates the hypothesis that the amount of growing hyphal tips positively affects protein secretion, and has important repercussions for industrial biotechnology.

## Results and discussion

### Apical distribution of secretory vesicles at hyphal tips is driven by secretory cargo in both wildtype and hyperbranching Δ*racA* strain

In order to study protein secretion in *A. niger* in a systematic manner, we selected our previously described reporter strain FG7 [30] (Table 1), which expresses the fluorescently tagged v-SNARE SncA (GFP-SncA) in an otherwise wildtype background as ancestor strain. In this strain, we deleted the chromosomal *glaA* gene, giving strain MF7.4. Western blot analysis of the culture supernatants of FG7 and MF7.4 cultivated in minimal medium (MM) supplemented with 5% w/v glucose confirmed that no glucoamylase was detectable in MF7.4 (Additional file 1: Fig. S1). Subsequently, we re-introduced the *glaA* gene into the *pyrG* locus but being this time under control of the doxycycline-inducible Tet-on expression system [33]. Correct integration of a single copy of Tet-on-*glaA* at *pyrG* in the resulting strain MF19.5 was confirmed by Southern analysis (Additional file 2: Fig. S2). This system enabled us to precisely control in a growth-independent manner *glaA* gene expression upon addition of doxycycline [33]. Finally, we deleted the endogenous *racA* gene in MF19.5 giving strain MF22.4. Respective cloning steps are described in detail in the “Methods” section. For brevity, we will further refer to FG7 as wildtype strain, MF7.4 as Δ*glaA* strain, MF22.4 as Δ*racA* strain and MF19.5 as the parental strain of MF22.4. As described above, all strains contain a single *egfp::sncA* gene copy and the distribution of post-Golgi carriers at hyphal tips can thus be directly compared among the strains.

**Table 1:** *Aspergillus niger* strains used in this work

Name	Genotype	Reference
FG7	∆*kusA, pyrG*^+^*, egfp::sncA* (derivative of MA70.15)	[30]
SS1.1	∆*kusA, pyrG*^−^*, egfp::sncA* (derivative of FG7)	This study
MF7.4	∆*kusA, pyrG*^+^*, egfp::sncA,* ∆*glaA::DR-AopyrG*-*DR* (derivative of SS1.1)	This study
MF9.1	∆*kusA, pyrG*^−^*, egfp::sncA,* ∆*glaA* (derivative of MF7.4)	This study
MF19.5	∆*kusA, pyrG*^+^*, egfp::sncA,*∆*glaA, Tet*-*on::glaA* (derivative of MF9.1)	This study
MF22.4	∆*kusA, pyrG*^+^*, egfp::sncA,* ∆g*laA, Tet*-*on::glaA, ∆racA::hygR* (derivative of MF19.5)	This study

**Fig. 1:**
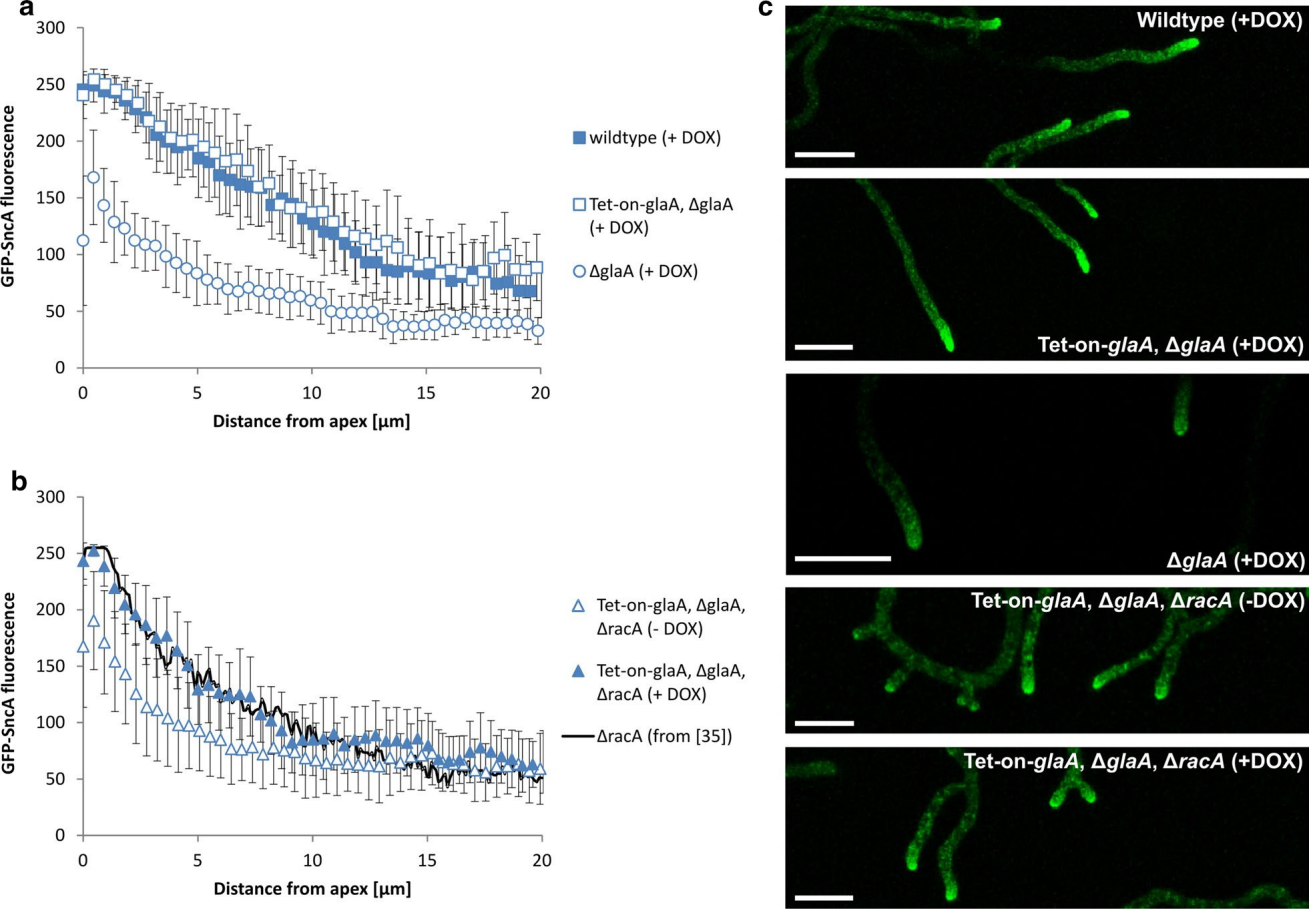
Distribution of secretory vesicles in both wildtype and hyperbranching (∆*racA*) backgrounds. Quantification of fluorescence intensity (arbitrary units) by CLSM microscopy of the post-Golgi vesicle marker SncA fused with GFP (**a**) in the wildtype (FG7), ∆*glaA* (MF7.4) and GlaA-overexpression (Tet-on-*glaA,* ∆*glaA*; MF19.5) strains with 20 µg/mL doxycycline (+DOX); (**b**) in the hyperbranching GlaA-overexpression strain (Tet-on-*glaA,* ∆*glaA*, ∆*racA*; MF22.4) with or without induction of *glaA* with DOX. All strains express the GFP-SncA fusion. Fluorescence of vesicles along the hyphae (up to 20 µm from the apex) was quantified from at least 20 hyphae. **c** Representative pictures (z-stacks) are shown (scale bar ca. 20 μm)

**Fig. 2:**
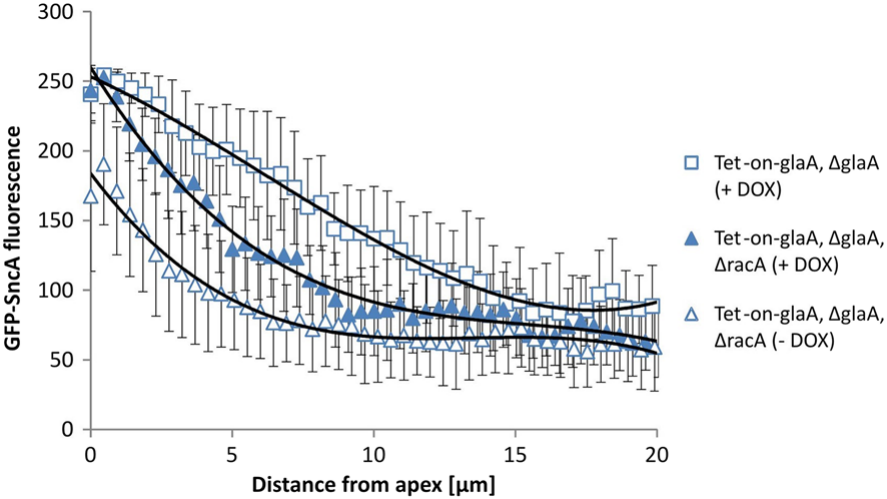
Polynomial curve approximation of distribution of secretory vesicles in both wildtype and hyperbranching (∆*racA*) backgrounds. Approximation of vesicle distribution for ∆*racA* (Tet-on-*glaA,* ∆*glaA*, ∆*racA*; MF22.4) and its parental strain (Tet-on-*glaA,* ∆*glaA*; MF19.5) under *glaA* overexpression conditions (+DOX) with a polynomial curve of the third order using an Excel trendline function. Both strains express the GFP-SncA fusion. Curves are taken from Fig. 1

All four strains were cultivated on minimal medium (MM) plates in the presence of glucose (known to induce glucoamylase expression) with or without 20 µg/mL doxycycline (DOX) for 2 days at 22 °C, and GFP-SncA fluorescence along 20 µm from the tip was quantified by confocal microscopy in at least 20 individual hyphae per strain (Fig. 1). As we reported before [35], the post-Golgi marker SncA shows a distribution with highest fluorescence at the near-apical region. Most interestingly, vesicle amount decreased significantly upon deletion of the chromosomal *glaA* gene, but, upon Tet-on driven *glaA* overexpression in strain MF19.5, a distribution of secretory vesicles identical to the wildtype was observed (Fig. 1a). These observations have four important implications. First, to the best of our knowledge it demonstrates for the first time that the transcriptional level of secretory cargoes indeed drives the amount and distribution of secretory vesicles at hyphal tips—i.e., if less protein molecules are destined for secretion, less secretory vesicles accumulate at hyphal tips. Second, it allows for the first time to obtain a rough estimation for the amount of post-Golgi secretory vesicles carrying protein cargoes mainly important for hyphal extension (73% = GFP-SncA fluorescence in the absence of DOX) as opposed to those destined for secretion. They account to a flexible capacity of up to 27%, which can be produced in response to increased transcription of protein cargo such as GlaA (Fig. 2). Third, it shows that transcription of *glaA* under control of the Tet-on systems or its own native promoter (the strongest known promoter in *A. niger* ensuring secretion of about 30 g/L glucoamylase into the environment [34]) gives comparable accumulation of secretory vesicles at hyphal tips. Fourth, it suggests that there is a maximum amount of secretory vesicles an individual hyphal tip can accommodate, as the GFP-SncA fluorescent profiles of hyphae from the wildtype strain (FG7) and the Tet-on-*glaA*, Δ*glaA* strain (MF19.5) upon presence of 20 µg/mL DOX perfectly overlapped.

We thus investigated the distribution of secretory vesicles in the hyperbranching Δ*racA* strain MF22.4 either with or without DOX (Fig. 1b) and could validate the observations formulated above: In the absence of *glaA* expression (−DOX condition), less secretory vesicles were visible at the hyphal tip. Upon *glaA* overexpression (+DOX condition), more secretory vesicles accumulated at hyphal tips. Notably, the latter vesicle distribution perfectly overlapped with data obtained previously upon native *glaA* expression in the Δ*racA* strain [35]; Fig. 1b), which not only reflects the reproducibility of this approach but again strongly implies that there is a maximum amount of post-Golgi carriers which can be accommodated by a growing hypha at the apex.

Remarkably, the amount of post-Golgi vesicles is reduced in the Δ*racA* mutant when compared to its parental strain (Fig. 1). The gradient is sharpened upon both native *glaA* expression [35] and Tet-on driven *glaA* overexpression (this study). As shown in Fig. 2, GFP-SncA fluorescence curves of the Δ*racA* mutant and its parental strain show a convex and concave shape, respectively, indicating that both amount and distribution of secretory vesicles differ in the two strains. We calculated the amounts of secretory vesicles along 20 µm hyphal tips by approximating the measured GFP-SncA fluorescence with a third order polynomial curve (for calculations, see Methods). This calculation indicated that the hyperbranching Δ*racA* strain has, along the first 20 µm of hyphal tips, on average ca. 23% less vesicles. We previously reported that deletion of *racA* results in about 30% higher branching frequency, and enumeration of hyphal apices in individual mycelia harvested from controlled bioreactor cultivations in Δ*racA* and parental strains resulted in 17 ± 6 for the wildtype (N402) and 22 ± 6 for the Δ*racA* mutant (Table 1 in [35]); that is, the wildtype has 77% of hyphal tips in comparison to the Δ*racA* mutant. This is in perfect agreement with the ratio calculated in this study.

The observations regarding the amount and distribution of secretory vesicles along hyphal tips again suggest that the total pool of post-Golgi secretory vesicles is similar in both strains but simply distributed to more hyphal tips in the Δ*racA* strain as we have previously proposed [35]. Furthermore, it implies that tip-directed secretion is somehow differently orchestrated in the Δ*racA* strain compared to the wildtype. This might be mechanistically explained by the previously observed shift of the endocytotic ring towards the hyphal apex (about 1–2 μm) of *A. niger* as visualised by the marker proteins AbpA (actin-binding protein involved in invagination, scission and release of endocytotic vesicles) and SlaB (adapter protein linking actin to endocytosis and involved in early endocytic site initiation; [35, 36]).

### Tet-on-driven *glaA* overexpression in Δ*racA* results in increased GlaA secretion

**Fig. 3:**
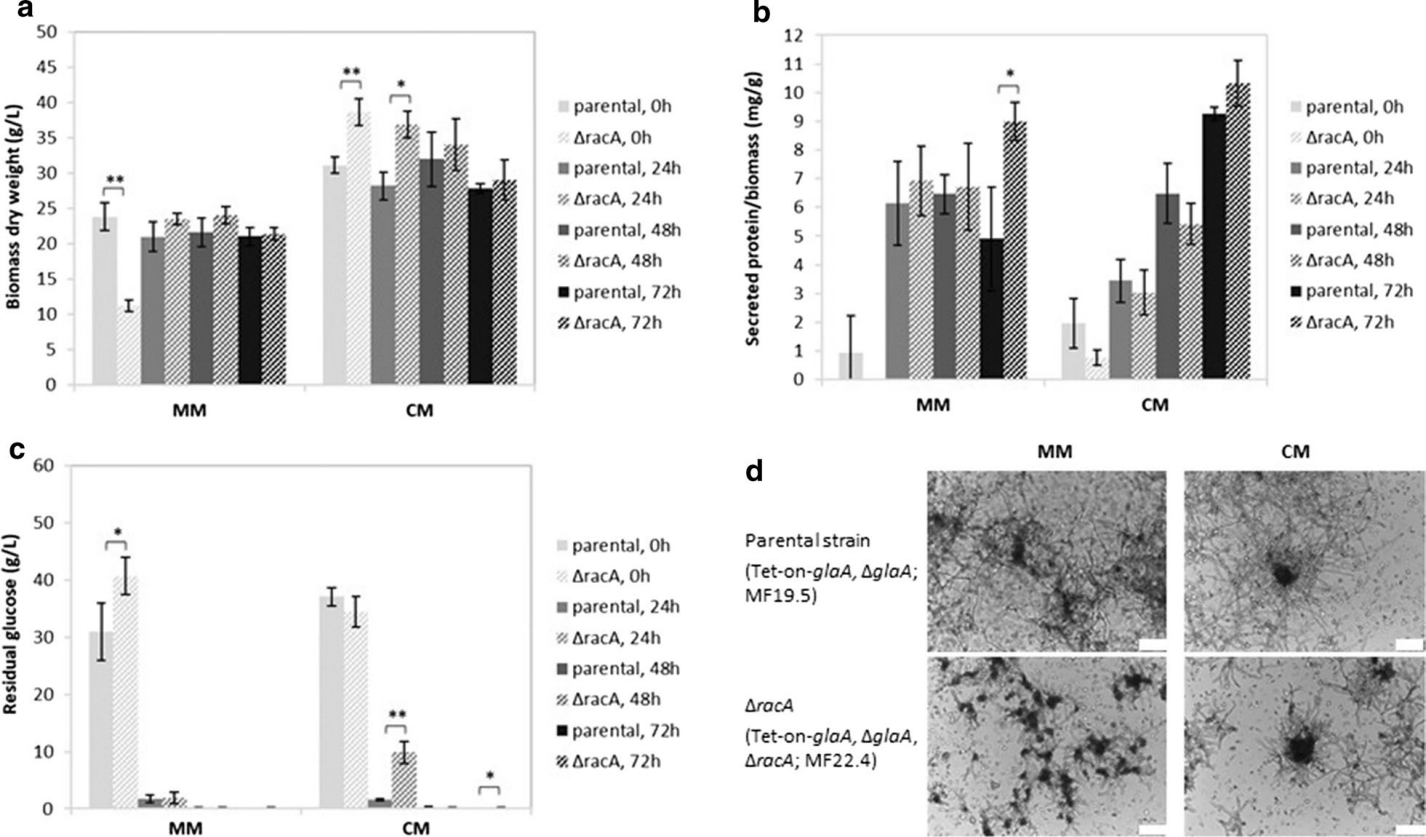
Growth profiles and protein secretion of both wildtype and hyperbranching (∆*racA*) backgrounds obtained from shake flask cultivations. The ∆*racA* (Tet-on-*glaA,* ∆*glaA*, ∆*racA;* MF22.4) and its parental strain (Tet-on-*glaA,* ∆*glaA*; MF19.5) were used in this experiment. For each strain, 5 × 10^6^ spores/mL were inoculated in 50 mL medium in Erlenmeyer flasks, cultivated for 18 h at 30 °C and 250 rpm. Glucoamylase production was induced with 20 µg/mL DOX (time point 0 h). 0, 24, 48 and 72 h post-induction, physiological parameters were determined and microscopic pictures taken. **a** Biomass yield (dry weight), **b** total secreted protein, and **c** residual glucose concentration in the media was determined. Results are average and error of three biological replicates. Significance values were calculated with 2-tailed t-test with independent variables (*p < 0.05, **p < 0.01). **d** Microscopic pictures showing representative pictures of mycelial macromorphologies at 0 h post-induction (scale bar 100 µm)

Strain MF19.5 (parental strain with Tet-on-*glaA,* Δ*glaA*) and MF22.4 (Tet-on-*glaA,* Δ*glaA,* Δ*racA*) were cultivated in 50 mL MM and complete medium (CM) for 18 h in the presence of microtalc particles to control mycelial macromorphologies [37]. Induction of *glaA* transcription was achieved by the addition of 20 µg/mL DOX, and physiological parameters were measured at 0, 24, 48 and 72 h post induction. Biomass yield and total protein secretion was overall similar in both strains after 24, 48 and 72 h (Fig. 3a, b). In agreement, total GFP-SncA fluorescence signals in freeze dried biomass samples of both strains did not differ at these time points (Additional file 3: Fig. S3). The exponential growth phase of both strains was concluded already after 48 h post induction, as glucose was completely consumed at this time point (Fig. 3c). We noted, however, that both strains differed in biomass accumulation and glucose consumption after 18 h of pre-cultivation (Fig. 3a, c), which suggested that the Δ*racA* strain consumes glucose more slowly. As a consequence, both strains might have entered the post-exponential growth phase at different time points. Microscopic analyses revealed dispersed macromorphologies for both strains which is due to the addition of microtalc particles (Fig. 3d). However, smaller and a bit more compact mycelial clumps which branched more frequent were, as expected, observed for the ∆*racA* strain. As their diameter was less than 200 μm in size (Fig. 3d), which is the critical transport distance for oxygen penetrating *A. niger* aggregates [38], any differences in mass transfer limitations are very unlikely.

**Fig. 4:**
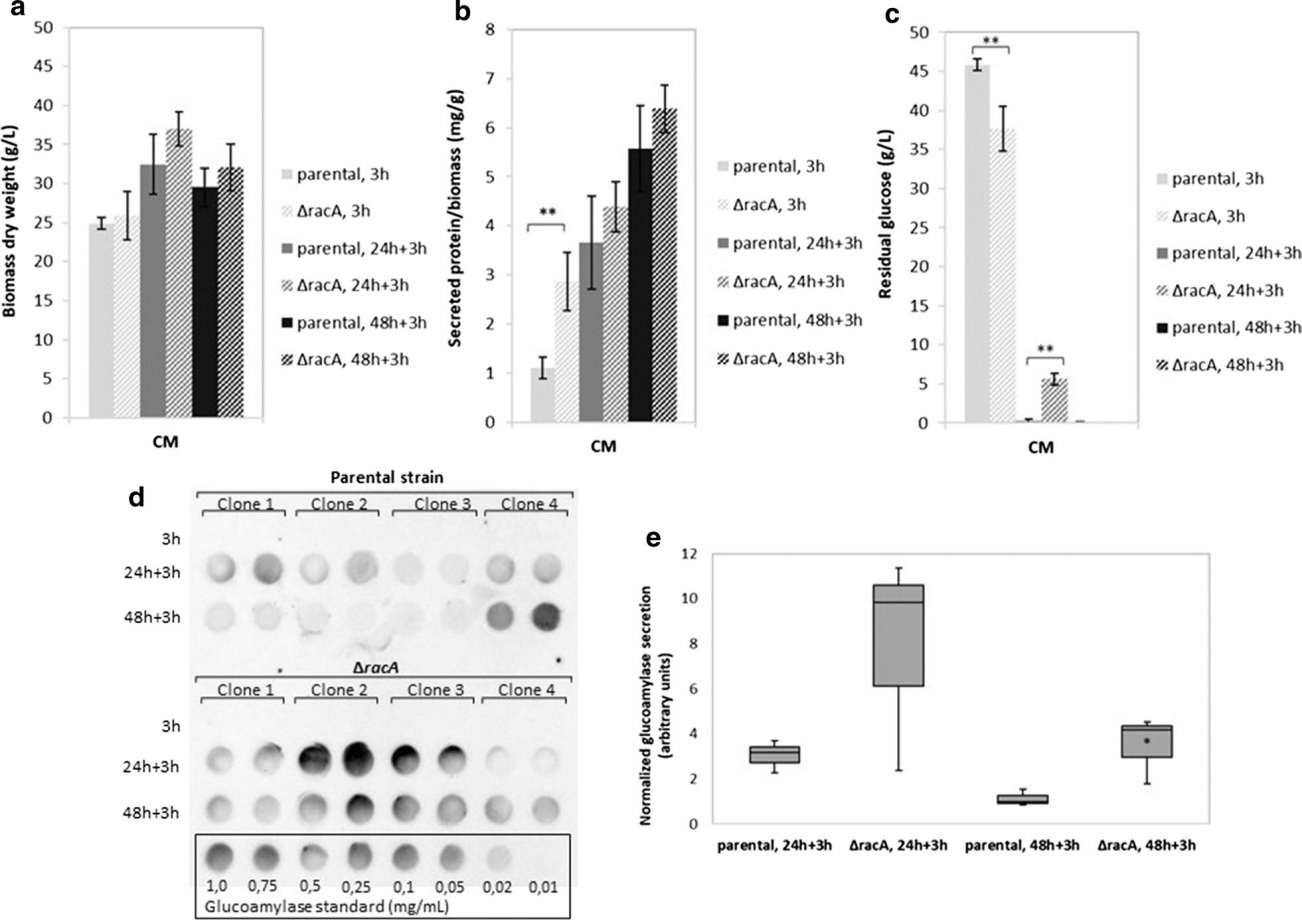
Growth profiles and protein secretion of both wildtype and hyperbranching (∆*racA*) backgrounds obtained from shake flask cultivations after repeated DOX induction. The ∆*racA* (Tet-on-*glaA,* ∆*glaA*, ∆*racA*; MF22.4) and its parental strain (Tet-on-*glaA,* ∆*glaA*; MF19.5) were pre-grown for 18 h as described in Fig. 3; glucoamylase production was then induced with 20 µg/mL DOX, as well as after additional 24 and 48 h; samples were collected 3 h post-induction with DOX to obtain time points 3 h, 24 + 3 h and 48 + 3 h, after which (**a**) biomass yield (dry weight), (**b**) total protein secretion and residual glucose (**c**) were determined as reported in Fig. 3. **d**, **e** Glucoamylase secretion was quantified by dot blot analysis using a monoclonal antibody. Each sample was spotted twice (technical replicate) and signal intensities quantified with ImageJ. Lowest row of the dot blot includes a dilution of glucoamylase as standard (0.01–1.00 mg/mL glucoamylase). Note the clonal variance between the four biological replicates, which is a general phenomenon in culture samples taken from shake flask cultivations. We decided to discarded clone 4 from both strains since we observed an inverse trend in comparison with the other three clones with regard to glucoamylase secretion (i.e. for clones 1–3 less extracellular GlaA was observed at time point 48 + 3 h than at 24 + 3 h, whereas for clone 4 the opposite was true). Results are thus calculated from the first three biological replicates. Average signal intensity of the technical duplicates was determined, and values were normalized by biomass yield and total protein secretion before calculating median values and quartiles, and plotting on box plots. Lowest median value (i.e. median for parental strain at 48 + 3 h) was set arbitrarily as 1. Significance values were calculated with 2-tailed (bar charts) or 1-tailed (box plots) t-test with independent variables (*p < 0.05, **p < 0.01)

As it is known that DOX stability is dependent on the pH and, on the other hand, growth and morphology of *A. niger* is unaffected by the addition of 125 μg/mL DOX [33], we decided to repeat the experiment described above but to add DOX repeatedly. In doing so, we cultivated biological quadruplicates of the parental and the ∆*racA* strain in CM plus microtalc particles in 50 mL liquid shake flask cultures with pulses of induction with 20 μg/mL DOX (after 18 h pre-incubation, considered as time point 0 h, as well as after additional 24 and 48 h). We ran the experiments in CM only to obtain higher biomass yields. We determined biomass yield, glucose consumption, total protein secretion and secreted GlaA in the supernatant by Western analysis (dot blot), each 3 h post induction (Fig. 4). Biomass yield and total protein secretion of both strains gave comparable results (except for higher values in total protein secretion for the ∆*racA* strain at time point 0 + 3 h). Remarkably, differences in glucose consumption became again apparent as already observed in the previous experiment. As shown in Fig. 4b, the ∆*racA* hyperbranching strain seemed to consume glucose faster prior to time point 0 + 3 h, but slower upon further cultivation (24 + 3 h). No detectable secreted GlaA 3 h after the first induction pulse with DOX demonstrates, as expected, that the Tet-on system is tight [33], and that longer incubation times are needed to achieve detectable levels of extracellular GlaA. Pairwise comparison of values for ∆*racA* and parental strain showed an up to 4-fold increase in glucoamylase secretion in the hyperbranching strain at time point 24 + 3 h and 48 + 3 h (*p* < 0.05). Less glucoamylase at time point 48 + 3 h in comparison with 24 + 3 h suggests extracellular degradation of the enzyme (the cultures must have entered post-exponential growth phase already prior to 48 h as glucose is fully consumed in both strains), and might also be due to differences in glucose consumption in both strains (Figs. 3c, 4c).

Based on the data presented here, we concluded that when a secretory cargo, which is not important for sustained hyphal tip extension, is increased by Tet-on driven overexpression, a ∆*racA* hyperbranching phenotype is advantageous to release more cargo, i.e. GlaA, into the medium. The transcriptomic fingerprint of *racA* loss-of-function uncovered that 139 out of 14,165 *A. niger* genes were differentially expressed [35], which likely form the fundamental basis for this observation. Besides genes predicted to encode proteins functioning in protein trafficking, actin localisation, (phospho)lipid metabolism and calcium signalling, also four genes supposedly related to carbon catabolism were differentially expressed: An06g00560 (ortholog of the *Saccharomyces cerevisiae* Hxt13p hexose transporter) and An12g00160 (ortholog of the *Saccharomyces cerevisiae* Mae1 malic enzyme) were both up-regulated, whereas An16g01770 (predicted xylose reductase) and An07g01340 (predicted phosphoenolpyruvate carboxylase) were both down-regulated in the ∆*racA* strain in comparison to the wildtype. It becomes therefore important to elucidate in future studies, whether the Tet-on driven positive effect on GlaA secretion is attributed to the hyperbranching phenotype only or additionally also linked to different metabolic activities in the ∆*racA* and its parental strain.

Enhanced specific protein yields in the ∆*racA* hyperbranching strain can have enormous repercussion not only for GlaA production but also for other enzymes produced by *A. niger* in industrial biotechnology [8, 39]. The strain ∆*racA* is especially suitable for industrial exploitation since it does not show any apparent difference in maximum specific growth rate compared to the wildtype strain [35]. Remarkable phenotypes of the ∆*racA* strain are, however, a hyperpolarisation of actin at the hyphal apex [32], a shift of the endocytic ring of 1–2 μm towards the hyphal apex [35], a convex instead of a concave distribution of post-Golgi secretory vesicles at the hyphal tip, even under Tet-on forced transcription of the *glaA* gene ([35] and this work) and a maximum level of post-Golgi secretory vesicles at an individual hyphal tip (this work). Possible explanations to bring these observations into a consistent framework could be that (i) the fusion kinetics of v-SNARE-labelled post-Golgi vesicles with the plasma membrane occurs faster in the ∆*racA* strain (convex GFP-SncA distribution), hence less post-Golgi vesicles accumulate at the tip and/or that (ii) post-Golgi vesicles which carried GlaA become much faster endocytosed, i.e. recycled due to the forward shift of the endocytic ring. In this context it is worth mentioning that different subpopulations of post-Golgi secretory vesicles have been described in *N. crassa*, where the Spitzenkörper consists of micro- and macrovesicles containing either chitin synthases, or glucan synthases, respectively [13, 40]. In *A. nidulans*, it was recently shown by superresolution microscopy that secretory vesicles containing the chitin synthase ChsB can be transported by kinesin-1 on microtubules very fast (7–10 μm/s) towards the tip and on early endosomes mediated by kinesin-3 much slower (2–7 μm/s) towards the hyphal tip and away from it (supposedly to the TGN; [16]). Hence, the v-SNARE SncA in *A. niger* could supposedly also localise to multiple vesicles and early endosomes and could also move with different velocities towards or away from the hyphal apex.

Interestingly, the overall amount of secreted proteins is identical in the wildtype and hyperbranching ∆*racA* strain (Figs. 3b, 4b), although the latter secreted about 4 times more GlaA (Fig. 4). Here, we speculate that this is likely balanced by the homeostatic RESS (repression under secretion stress) control system, a phenomenon well known for *A. niger*, which ensures a selective down-regulation of genes coding for extracellular enzymes when others are strongly up-regulated [41]. Still, one puzzling question remains to be answered in future experiments. Although the hyphal tip remains the main route of protein secretion in *A. niger*, how much of GlaA becomes released into the medium via septal secretion? In *A. oryzae*, septum-directed secretion of α-amylase (AmyB) has been shown [42], while *N. crassa* integrates the vacuolar pump PMA-1 into the plasma membrane subapically without passing the Spitzenkörper [43]. Both observations indicate alternative routes of secretion at non-apical hyphal regions. We most recently obtained supporting indications for GlaA accumulation at septal regions in *A. niger* [44]. It will be therefore interesting to study in the future, whether there is any higher GlaA secretion via septa in the ∆*racA* strain due to Tet-on driven overexpression of the *glaA* gene or not.

## Conclusions

In this study, we successfully validated the hypothesis that challenging the ∆*racA* strain to overexpress the *glaA* gene increases the amount of post-Golgi secretory vesicles at hyphal tips, and eventually results in up to 4-times higher secreted GlaA. Therefore, a positive correlation between the amount of growing hyphae and secretion exists in *A. niger*, given that transcription of the secretory protein is continuously forced by the Tet-on system. Given the enormous importance of *A. niger* as industrial cell factory for the production of proteins, enzyme and metabolites, this study has profound implications for biotechnology. Based on the data obtained in this study, we propose a *racA* deletion background as a default, hypersecretion strain for enhanced extracellular product yield.

## Methods

### Strains and general cloning procedures

Strains used in this study are summarized in Table 1, plasmids and primers in Additional file 4: Table S1. Molecular techniques for *E. coli* followed protocols described earlier [45]. *A. niger* transformation and genomic DNA extraction from selected transformants was done according to [46]. Strains were grown at 30 °C in minimal medium (MM) [47] or complete medium (CM), consisting of MM supplemented with 1% w/v yeast extract and 0.5% w/v casamino acids. When required, 100 µg/mL hygromycin, 10 mM uridine or 10 mM histidine were added to the medium.

To obtain *pyrG*^−^ strains, 2 × 10^7^ spores were plated on MM plates containing 75 mg/mL 5-fluoroorotic acid (FOA), 10 mM uridine and 10 mM proline. Plates were incubated at 30 °C for 1–2 weeks until single colonies were visible. FOA-resistant mutants were purified on MM + FOA plates once and tested for their uridine auxotrophy on MM plates or MM plates containing 10 mM uridine, respectively. Cloning and related molecular techniques were performed according to standard procedures [45], whereas *A. niger* transformation, genomic DNA extraction and Southern blot were performed as previously described [46].

Strain FG7 was used as ancestor strain in which the *pyrG* gene was counterselected with FOA to obtain the *pyrG*^−^ strain SS1.1. To construct a *pyrG*-recyclable *glaA* deletion cassette, promoter (P) and terminator (T) regions of the *glaA* gene were amplified using primers listed in Additional file 4: Table S1. Using a combined fusion PCR and ligation approach, a *PglaA*-*AopyrG*-*PglaA*-*TglaA* cassette was constructed and cloned in pJET1.2 giving rise to pSS3.34. This plasmid was transformed into strain SS1.1 to delete the *glaA* gene, giving strain MF7.4, which was selected via Southern analysis. The *AopyrG* marker was counterselected in this strain via FOA selection and strain MF9.1 was generated, being again uracil-auxotroph (*pyrG*^−^). To construct a *Tet*-*on::glaA* expression plasmid, the *glaA* sequence was amplified using primers listed in Additional file 4: Table S1 and ligated into the unique *PmeI* restriction site of the plasmid pVG2.2 [33] harbouring the Tet-on system and the A. niger pyrG* as selection marker, giving rise to pMF19.1. This construct was targeted to the *pyrG* locus in MF9.1 and strain MF19.5 was selected on transformation plates lacking uridine and verified via Southern analysis. To construct a *racA::hygR* deletion cassette for knock-out of the endogenous *racA* gene in MF19.5, the split marker approach was used [48]. In brief, the 5′ and 3′ sequences of *racA* and the hygromycin resistance gene were amplified using primers listed in Additional file 4: Table S1, fused via PCR, and ligated into the plasmid pJET1.2 giving rise to pMF14.3 (*PracA*-*hygR*) and pMF15.1 (*hygR::TracA*), respectively. Both fragments were transformed into strain MF19.5 and a transformant with a deleted *racA* gene was selected via Southern analysis (strain MF22.4).

### Confocal microscopy of individual *A. niger* hyphae

Microscopy was performed as previously described [30, 35]. Briefly, for confocal laser scanning microscopy (CLSM) conidia were spotted on MM plates, supplemented with different concentrations of doxycycline as indicated and incubated at 22 °C for 2 days, following excision of the colony and placing it upside down into a glass-bottom Petri dish. Liquid MM medium (if needed, supplemented with the same concentration of doxycycline that was present in the MM plate) was added and cells were incubated at 22 °C until the cells resumed growth. Cells were analysed using an inverted TCS SP8 confocal microscope system (Leica, Germany). Images were captured using a HC PL APO CS2 20×/0.75 IMM objective with a pinhole at airy unit 1 (48.8 µm) at an image resolution of 1024 × 1024 pixels at 700 Hz. For GFP detection, 3% laser (488 nm) intensity was used coupled with an emission detection of 495–545 nm at a gain of 800. 10 z-stacks were taken using the system-optimized calculation of z-stacks. The GFP-SncA fluorescence of single z-stacks was quantified with the provided software LAS X (Leica, Germany) using the tool “Draw Line” in the tab “Quantify”. A line was drawn by hand along the hyphae, starting from the tip, resulting in a value for the intensity of fluorescence (in arbitrary units, whereas 256 is the maximum value below overexposure) approximately every 0.46 µm. The fluorescence signal was measured over a length of 20 µm.

### Calculation of GFP-SncA fluorescence at hyphal tips

We calculated the amounts of secretory vesicles along 20 µm hyphal tips based on the measured GFP-SncA fluorescence. Fluorescent signal curves depicted in Fig. 2 were approximated using Excel (Microsoft Office Package 2010) with the following polynomial functions and coefficients of determination $R^{2}$

$$\left( {{\text{parental }}\;+\;{\text{DOX}}}\right)y\;=\;0.0311x^{3}\; - \;0.5712x^{2}\; - \;9.0959x\;+\;253.15\,({\text{with}}\,R^{2}\;=\;0.9902)$$

$$(\Delta racA + {\text{DOX}})y = -\;0.0432x^{3}\;+\; 1.9981x^{2}\;-\;32.4820x\;+\;259.68\;({\text{with}}\;R^{2}\;=\; 0.9859)$$

$$(\Delta racA - {\text{DOX}})y = - \;0.0499x^{3}\; + \;2.0248x^{2} \;-\; 26.9690x \;+\; 183.65\,({\text{with}}\,R^{2} = 0.9713)$$

Solving the polynomial functions over the whole 20 µm hyphal length with the integral

$$I = \mathop \int \limits_{0 \mu m}^{20 \mu m} \left( {ax^{3} + bx^{2} + cx + d} \right)dx$$

gives the following values (*I* = approximated amounts of vesicles along 20 µm hyphal tips)

$$\left( {{\text{parental }} + {\text{DOX}}} \right)\quad I = 2964.62$$

$$(\Delta racA + {\text{DOX}})\quad I = 2297.47$$

$$(\Delta racA - {\text{DOX}})\quad I = 1682.67$$

and the ratios

$$\frac{{\Delta racA + {\text{DOX}}}}{{{\text{parental}} + {\text{DOX}}}} = \frac{2297.47}{2964.62} \times 100 \% \cong 77 \%$$

$$\frac{{\Delta racA - {\text{DOX}}}}{{\Delta racA + {\text{DOX}}}} = \frac{1682.67}{2297.47} \times 100 \% \cong 73 \%$$

### Shake flask cultivations of *A. niger*

For production of glucoamylase, 5 × 10^6^ spores/mL of strains MF19.5 (Tet-on-*glaA,* ∆*glaA*) or MF22.4 (Tet-on-*glaA,* ∆*glaA*, ∆*racA*) were inoculated in 50 mL liquid medium and strains were cultivated at 30 °C, 250 rpm in shake flask cultures containing MM or CM with 5% w/v glucose as carbon source and with 10 g/L micro talc particles to control mycelial macromorphologies as described in [37]. For medium composition please refer to [46]. After 18 h incubation (considered as time point 0), Tet-on driven expression of GlaA was induced with 20 µg/mL doxycycline (DOX) and further incubated for 0, 24, 48 and 72 h before analysis of physiological parameters (biomass dry weight as well as total protein secretion and residual glucose concentration in the media; see below). For repeated induction of *glaA* expression in 50 mL liquid shake flask cultures of CM with 10 g/L micro talc particles, 20 µg/mL DOX was added 18 h post-inoculation (considered as time point 0) as well as after further 24 and 48 h of incubation. Samples were taken 3 h post-induction with DOX to determine physiological parameters and extracellular GlaA. For microscopic pictures, a small amount of culture (ca. 0, 1 mL) was sampled; images were taken using a SA8APO equipped with a MC120HD camera (Leica, USA). Experiments were performed as biological triplicates/quadruplicates.

### Determination of biomass dry weight, total protein secretion, residual glucose and total GFP-SncA fluorescence

From shake flask cultures, 4 mL of samples were taken at the indicated time point. Biomass and culture supernatant were separated by suction filtration under vacuum. Biomass was collected, frozen at − 80 °C, and freeze dried overnight to determine biomass yield (dry weight). Total protein secretion in the culture supernatant was determined via the Bradford assay (BioRad) according to the manufacturers’ protocols and absorbance (600 nm) was measured using a GloMax^®^-Multi Detection System (Promega). Quantification of residual glucose in the cultivation medium was performed with the Glucose GOP/PAP Liquicolor kit (Human, Germany) according to the manufacturer’s manual. Total GFP-SncA fluorescence was determined in freeze dried biomass. 50 mg dried biomass were grinded and resuspended in 1 mL 50 mM NaPO_4_ buffer pH 7.0. Following ultrasonification for 10 min, fluorescence signal in supernatant was determined using a GloMax^®^-Multi Detection System (Promega) equipped with a blue filter (excitation: 490 nm, emission: 510–570 nm).

### Analysis of extracellular glucoamylase (GlaA) by Western analysis/dot blot

Supernatant harvested by suction filtration (see above) was analysed with regard to GlaA content by dot blot using the Minifold I Spot-Blot system (Whatman Schleicher & Schuell). Briefly, 100 µL samples were mixed with 150 µL phosphate buffer saline (PBS: 137 mM NaCl, 2.7 mM KCl, 1 mM Na_2_HPO_4_, 0.2 mM KH_2_PO_4_), heated at 100 °C for 10 min and cooled down. 200 μL were then spotted in the slots of the dot blot device provided with a nitrocellulose membrane (PROTRAN, Schleicher & Schuell) previously soaked on PBS under vacuum to allow protein binding. After suction, 200 μL PBS were applied to wash the membrane under vacuum. A standard dilution of GlaA (0.01–1.00 mg/mL glucoamylase) was done in PBS and blotted as described above. The membrane was then removed from the dot blot device and soaked 1 h at 25 °C in 30 mL Protein Blocking Buffer (PBB: 100 mg/mL milk powder in PBS + 0.1% v/v Tween 20) under shaking. Monoclonal anti-GlaA antibody (kindly provided by Peter Punt, TNO, The Netherlands) was then added (10 µL, i.e. final dilution 1:3000), and the membrane was further incubated overnight at 4 °C under shaking. PBB was discarded and membrane washed three times for 5 min at 25 °C with PBS + 0.1% v/v Tween 20. HRP-conjugated, secondary anti-mouse antibody (Agilent Technologies, USA) was then added (6.7 µL in 20 mL PBB, i.e. final dilution 1:3000), and the membrane was incubated for 1 h at 25 °C under shaking. PBB was discarded, and membrane washed (three times with PBS + 0.1% v/v Tween 20, and then once with PBS for 5 min each at 25 °C). Chemiluminescence reaction was performed by using an ECL Prime Western Blotting Detection Kit (GE Healthcare), and signal detected with ChemiDoc™ MP Imaging System using the Image Lab software (both from BioRad). Signal intensity was quantified with the open source software ImageJ using a standard protocol (https://imagej.nih.gov/ij/docs/examples/dot-blot/).

For Western blot analysis (Additional file 4: Fig. S1), 10 µL culture supernatant of FG7 (wildtype) and MF7.4 (∆*glaA*) grown in MM in 20 mL shake flask culture for 90 h at 30 °C, 250 rpm (inoculation 5 × 10^6^ spores/mL) were loaded to a 12% w/v SDS-PAGE and transferred to a PVDF membrane (Roth, Germany) after the proteins have been separated. Detection was performed with the same primary and secondary antibodies as described above. 10 µL of culture supernatant were directly analysed via Western blot using an anti-glucoamylase antibody. Incubations were performed in PBS + 0.1% v/v Tween 20 supplemented with 5% w/v dry milk. The primary antibody incubation was performed at 4 °C for 16 h, while the blot was incubated with the secondary antibody at room temperature for 1 h. Chemiluminescence reaction was performed by using an ECL Prime Western Blotting Detection Kit (GE Healthcare).

## Additional files

**Additional file 1: Fig. S1.** Western blot analysis of wildtype (FG7) and ∆*glaA* (MF7.4). 5 × 10^6^ spores/mL were inoculated in 20 mL MM medium in Erlenmeyer flasks, and cultivated for 18 h at 30 °C and 250 rpm. 10 µL of culture supernatant were directly analysed via Western blot using an anti-glucoamylase antibody.

**Additional file 2: Fig. S2.** Southern blot analysis of wildtype (N402) and Tet-on-*glaA* (MF19.5). The *glaA* gene under control of the doxycycline-inducible Tet-on expression system was re-introduced into the *pyrG* locus of MF9.1, resulting in the Tet-on-*glaA* strain MF19.5. Correct integration of a single copy at *pyrG* was confirmed by Southern blotting (A). Genomic DNA of MF19.5 and N402 was digested using *NcoI* and *BsrGI*. and hybridised with a 600 bp probe, homologous to parts of the *pyrG* gene. The expected band sizes were 9005 bp + 4231 bp for MF19.5 and 3126 bp for N402, respectively (B).

**Additional file 3: Fig. S3.** Total GFP-SncA fluorescence in freeze dried biomass of both wildtype and hyperbranching (∆*racA*) backgrounds obtained from shake flask cultivations. The ∆*racA* (Tet-on-*glaA,* ∆*glaA*, ∆*racA;* MF22.4) and its parental strain (Tet-on-*glaA,* ∆*glaA*; MF19.5) were used in this experiment. Each 5 × 10^6^ spores/mL were inoculated in 50 mL medium in Erlenmeyer flasks, cultivated for 18 h at 30 °C and 250 rpm. Glucoamylase production was induced with 20 µg/mL DOX (time point 0 h). 0, 24, 48 and 72 h post-induction, biomass was collected and freeze dried. Total GFP-SncA fluorescence was determined in 50 mg freeze dried biomass. Results are average and error of three biological replicates. Significance values were calculated with 2-tailed t-test with independent variables (*p < 0.05, **p < 0.01).

**Additional file 4: Table S1.** Plasmids and primers used in this work.
