# Supplementary figures and images for: Construction of an improved Aspergillus niger platform for enhanced glucoamylase secretion
Source: Microb Cell Fact. 2018 Jun 16;17:95. doi: 10.1186/s12934-018-0941-8 (PMC6004097; doi:10.1186/s12934-018-0941-8)

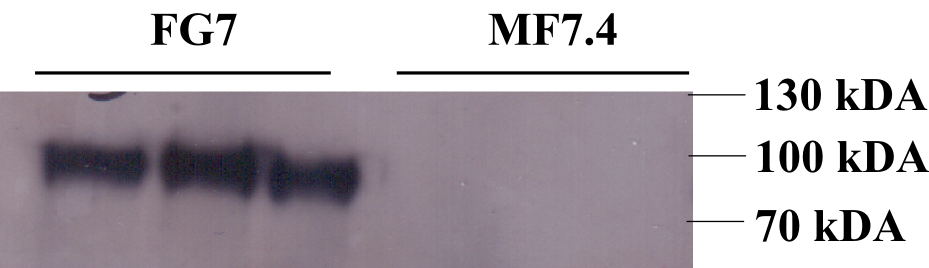

Supplement: Supplementary file 1 — Additional file 1: Fig. S1. Western blot analysis of wildtype (FG7) and ∆glaA (MF7.4). 5 × 10^6^ spores/mL were inoculated in 20 mL MM medium in Erlenmeyer flasks, and cultivated for 18 h at 30 °C and 250 rpm. 10 µL of culture supernatant were directly analysed via Western blot using an anti-glucoamylase antibody. [file 12934_2018_941_MOESM1_ESM.png]

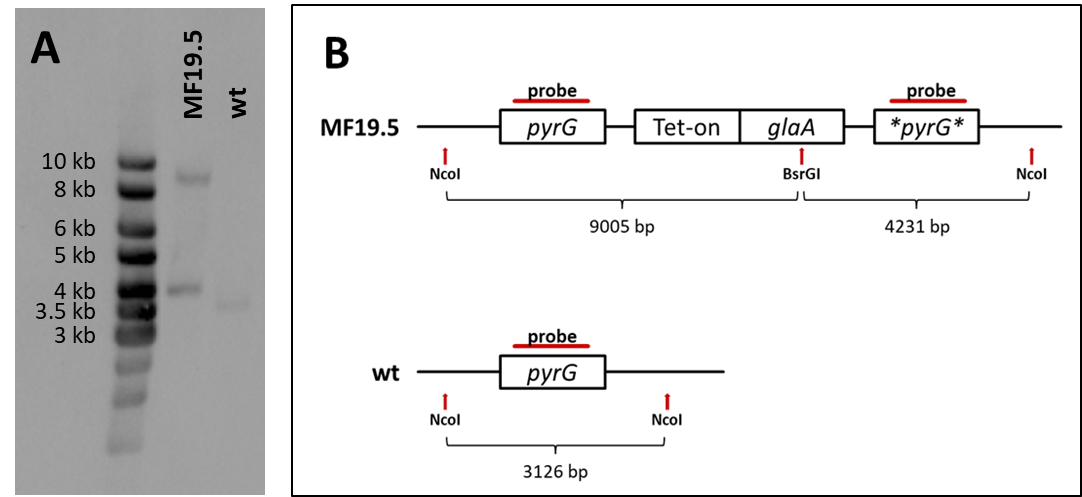

Supplement: Supplementary file 2 — Additional file 2: Fig. S2. Southern blot analysis of wildtype (N402) and Tet-on-glaA (MF19.5). The glaA gene under control of the doxycycline-inducible Tet-on expression system was re-introduced into the pyrG locus of MF9.1, resulting in the Tet-on-glaA strain MF19.5. Correct integration of a single copy at pyrG was confirmed by Southern blotting (A). Genomic DNA of MF19.5 and N402 was digested using NcoI and BsrGI. and hybridised with a 600 bp probe, homologous to parts of the pyrG gene. The expected band sizes were 9005 bp + 4231 bp for MF19.5 and 3126 bp for N402, respectively (B). [file 12934_2018_941_MOESM2_ESM.png]

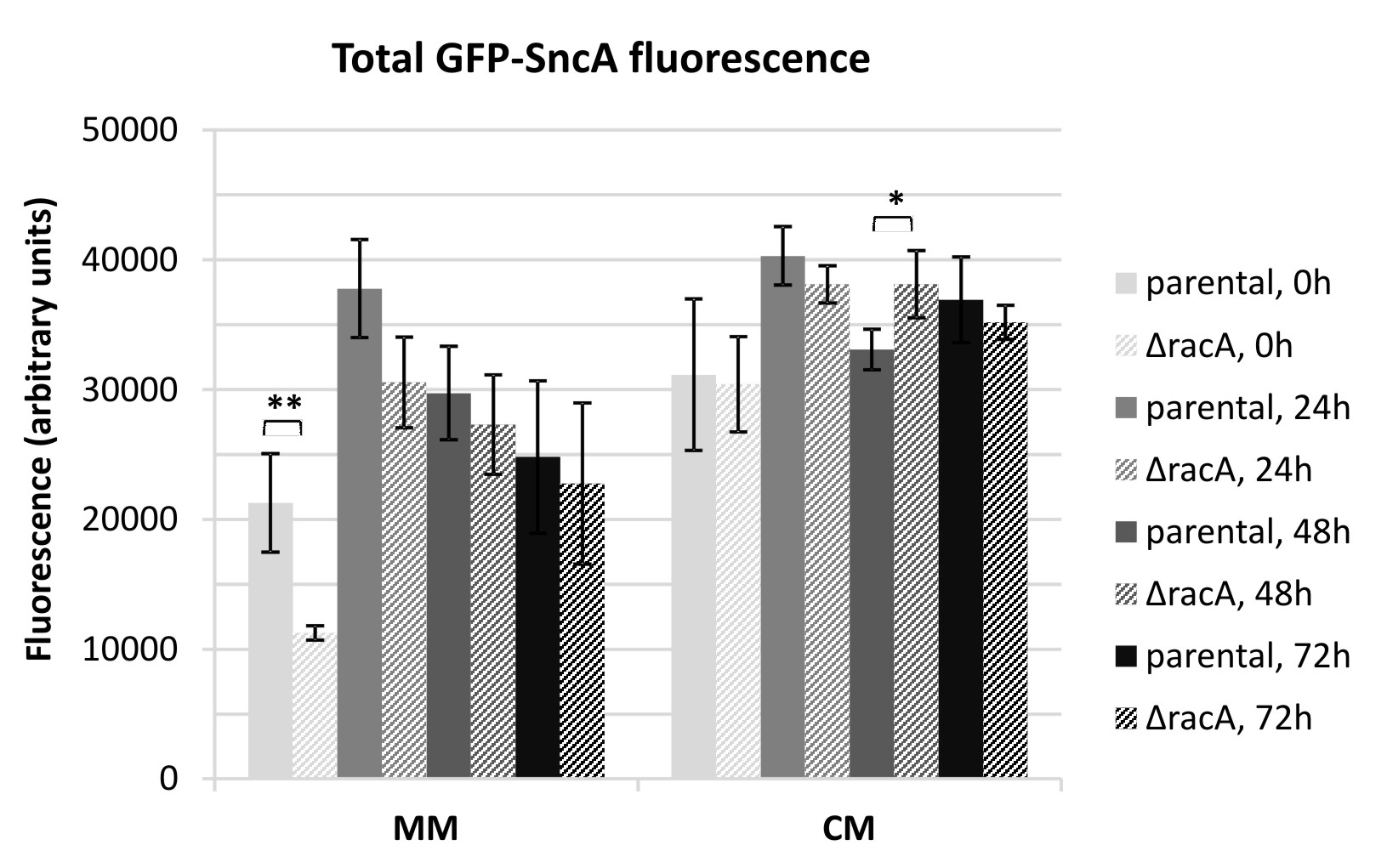

Supplement: Supplementary file 3 — Additional file 3: Fig. S3. Total GFP-SncA fluorescence in freeze dried biomass of both wildtype and hyperbranching (∆racA) backgrounds obtained from shake flask cultivations. The ∆racA (Tet-on-glaA, ∆glaA, ∆racA; MF22.4) and its parental strain (Tet-on-glaA, ∆glaA; MF19.5) were used in this experiment. Each 5 × 10^6^ spores/mL were inoculated in 50 mL medium in Erlenmeyer flasks, cultivated for 18 h at 30 °C and 250 rpm. Glucoamylase production was induced with 20 µg/mL DOX (time point 0 h). 0, 24, 48 and 72 h post-induction, biomass was collected and freeze dried. Total GFP-SncA fluorescence was determined in 50 mg freeze dried biomass. Results are average and error of three biological replicates. Significance values were calculated with 2-tailed t-test with independent variables (*p < 0.05, **p < 0.01). [file 12934_2018_941_MOESM3_ESM.jpg]
