# Supplementary material for: Construction of an improved Aspergillus niger platform for enhanced glucoamylase secretion
Source: Microb Cell Fact. 2018 Jun 16;17:95. doi: 10.1186/s12934-018-0941-8 (PMC6004097; doi:10.1186/s12934-018-0941-8)
Supplement: Supplementary file 4 — Additional file 4: Table S1. Plasmids and primers used in this work. [file 12934_2018_941_MOESM4_ESM.docx]

**Additional Table S1.** Plasmids and primers used in this work.

| **Construct** | **Primer** | **Sequence (5’ to 3’ oriented)** | **Target** | **ORF code** |
| --- | --- | --- | --- | --- |
| pSS3.34 | PglaA_NotI_fw | tttgcggccgcCTGGATCCGAACTCCAACC | *5' glaA* | An03g06550 |
|  | PglaA_pyrGfurev | gcacttaccttcgcatttAGCACTTCAGCCCCTCAC |  |  |
|  | PyrG_PglaAfu_fw | gggctgaagtgctAAATGCGAAGGTAAGTGCTTCT | *AOpyrG* | AO090011000868 |
|  | PyrG_SalI_rev | atatgtcgacGCCTCATTTCCCACAGGTT |  |  |
|  | PglaA_shortSalI | aatgtcgacGGACCTGCGTTATAGCTTCC | *5' glaA* | An03g06550 |
|  | PglaA_short_Tfu | atccccatcctttaacAGCACTTCAGCCCCTCAC |  |  |
|  | TglaA_fu_fw | ggggctgaagtgctGTTAAAGGATGGGGATGAGG | *3' glaA* | An03g06550 |
|  | TglaA_HpaI_rev | catgttaacAATCTCCCTTGCACGATGTT |  |  |
| pMF14.3 | PracA_fw | AGCAGCAGCAGCAACACTAA | *5' racA* | An11g10030 |
|  | Pracfurev | tctctctgcgtccgtATTGAGGCGAGGGATGG |  |  |
|  | HpHfufwnew | ccctcgcctcaatACGGACGCAGAGAGAAGG | *hygR* |  |
|  | pHygrev | GAAATTGCCGTCAACCAA |  |  |
| pMF15.1 | TracAfufwnew | gcagatcaacggtcgGGCCAAACCGAAGAACA | *3' racA* | An11g10030 |
|  | TracA_rev | CAACTACGACCGCATGAAGA |  |  |
|  | tHygfw | AGAGCCTGACCTATTGCATCT | *hygR* |  |
|  | HpHfurevnew | tcttcggtttggccCGACCGTTGATCTGCTTG |  |  |
| pMF19.1 | tetnew_glaA_fw | taccccgcttgagcagacatcaccgtttaaacaccATGTCGTTCCGATCTCTACTCG | *glaA* | An03g06550 |
|  | GlaA_tet_rev | ataagtttaaacCACATGACTTGGCTTCCATTT |  |  |
